# Description of two new Ecuadorian *Zilchistrophia* Weyrauch, 1960, with the clarification of the systematic position of the genus based on anatomical data (Gastropoda, Stylommatophora, Scolodontidae)

**DOI:** 10.3897/zookeys.453.8605

**Published:** 2014-11-10

**Authors:** Barna Páll-Gergely, Takahiro Asami

**Affiliations:** 1 Department of Biology, Shinshu University, Matsumoto 390-8621, Japan

**Keywords:** Systrophiidae, Plectopylidae, *Plectopylis*, Corillidae, anatomy, taxonomy

## Abstract

Two new species of the genus *Zilchistrophia* Weyrauch, 1960 are described from Eastern Ecuadorian rain forest: *Zilchistrophia hilaryae* **sp. n.** and *Zilchistrophia shiwiarorum* **sp. n.** These two new species extend the distribution of the genus considerably northwards, because congeners have been reported from Peru only. For the first time we present anatomical data (radula, buccal mass, morphology of the foot and the genital structure) of *Zilchistrophia* species. According to these, the genus belongs to the family Scolodontidae, subfamily Scolodontinae (=“Systrophiini”). The previously assumed systematic relationship of *Zilchistrophia* with the Asian Corillidae and Plectopylidae based on the similarly looking palatal plicae is not supported.

## Introduction

In the collection of the Natural History Museum, London, we encountered some shells and ethanol-preserved specimens collected in Eastern Ecuador during an expedition organized by The Shiwiar Rainforest Initiative 2000. Some of these specimens represent two species new to science. The small (3.5–5 mm), translucent, flat shells possess two or three horizontal palatal plicae standing one above the other, approximately a third to a half whorl behind the aperture. Similar species have been reported from Peru under the name *Zilchistrophia* Weyrauch, 1960. Weyrauch (1960) created the genus for three species: *Zilchistrophia tridentata* Weyrauch, 1960 (type species by original designation), Systrophia (Systrophia) obvoluta Haas, 1949 and Systrophia (Systrophia) angigyra Haas, 1949. Although the two Ecuadorian new species differ somewhat from the Peruvian ones in terms of the formation of the last quarter of whorl, we classify them as “true” *Zilchistrophia* species, and use the information on their soft anatomy to clarify the taxonomic status of *Zilchistrophia*.

## Material and methods

The two new Ecuadorian species were compared with the holotype of *Zilchistrophia tridentata* (“C-Peru, Pichita Caluga, 2200 m, im Canchamayo-Becken”, leg. Weyrauch 18.08.1959., SMF 162006), and the original descriptions and photos of the other two Peruvian species. Ethanol-preserved specimens were dissected under Leica stereomicroscope, a camera on which provided photographs. To describe the reproductive system, we used the terms “proximal” and “distal” in relation to the centre of the body.

The buccal mass was removed and soaked in 2 molar KOH solution for 5 hours before extracting radula, which was preserved in 70% ethanol. Radulae were directly observed without coating under a low vacuum SEM (Miniscope TM-1000, Hitachi High-Technologies, Tokyo).

The nomenclature of plicae follow Páll-Gergely and Hunyadi (2013): horizontal folds (=parallel with the suture) are called plicae, whereas vertical folds (=perpendicular to the suture) are named lamellae.

The geographical coordinates of localities mentioned in this paper are the following: Chuintsa 02°00.891'S, 076°40.866'W; Nuevo Corrientes 01°59.870'S, 076°45.968'W.

### Abbreviations

D shell diameter

H shell height

NHMUK The Natural History Museum (London, United Kingdom)

SMF Senckenberg Forschungsinstitut und Naturmuseum (Frankfurt am Main, Germany)

## Taxonomic descriptions

### Scolodontidae

Taxon classificationAnimaliaStylommatophoraScolodontidae

Baker, 1925

Scolodontidae Baker, 1925, The Nautilus 38(3): 88.

#### Type genus.

*Scolodonta* Döring, 1875.

#### Remarks.

For taxonomic and nomenclatural notes see Hausdorf (2006).

### Genus Zilchistrophia

Taxon classificationAnimaliaStylommatophoraScolodontidae

Weyrauch, 1960

Zilchistrophia Weyrauch, 1960, Archiv für Molluskenkunde 89 (1/3): 26.

#### Type species.

*Zilchistrophia tridentata* Weyrauch, 1960, by original designation.

### Zilchistrophia hilaryae

Taxon classificationAnimaliaStylommatophoraScolodontidae

Páll-Gergely sp. n.

http://zoobank.org/6A755D8B-809F-4FCB-B23B-DC9A82D28FEE

**Figure 1.**
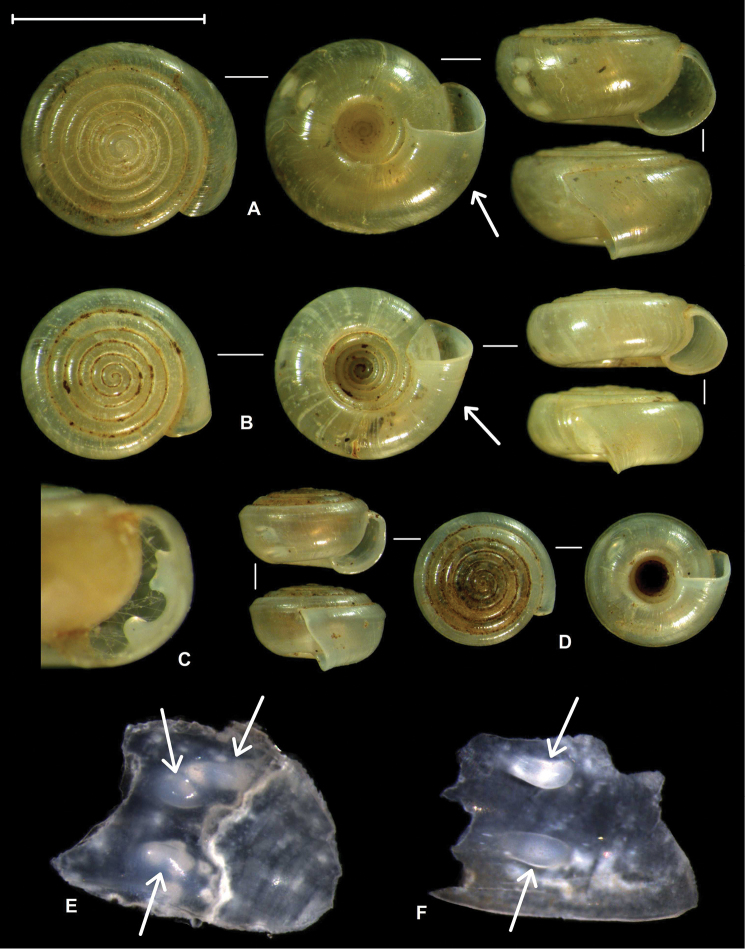
Shells of *Zilchistrophia* Weyrauch, 1960 species. **A** Holotype of *Zilchistrophia tridentata* Weyrauch, 1960 (SMF 162006; type species of the genus), arrow shows the inflated part of the last whorl **B** holotype of *Zilchistrophia hilaryae* sp. n. (NHMUK 20020375.1), arrow shows the non-inflated part of the body whorl **C** paratype of *Zilchistrophia hilaryae* sp. n. (subadult shell with the last quarter of whorl removed in order to show palatal plicae) **D** holotype of *Zilchistrophia shiwiarorum* sp. n. (NHMUK 20020382) **E** plicae bearing shell fragment of the anatomically examined specimen of *Zilchistrophia hilaryae* sp. n. (arrows indicate the plicae) **F** plicae bearing shell fragment of the anatomically examined specimen of *Zilchistrophia shiwiarorum* sp. n. (arrows indicate the plicae). The two shell fragments (**E** and **F**) are left together with the ethanol-preserved body. Scale represents 5 mm, and refers to **A, B** and **D**.

**Figure 2.**
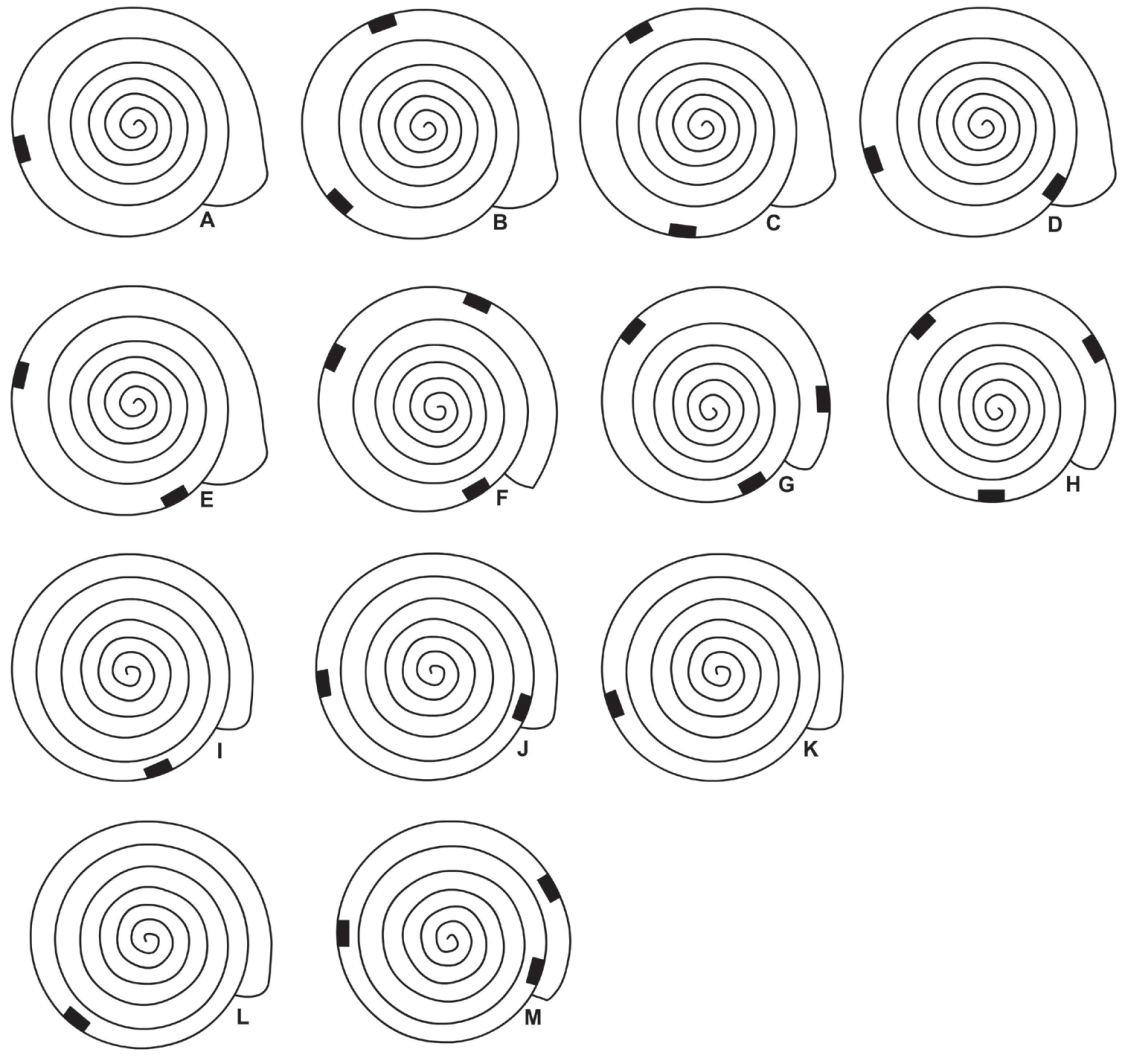
Schematic drawings showing the position of plicae sets in *Zilchistrophia hilaryae* sp. n. (**A–H**) and *Zilchistrophia shiwiarorum* sp. n. (**I–M**). Adult shells: **A–E, I–L**; juvenile shells: **F–H, M. A** NHMUK 20020375.1 (holotype) **B** NHMUK 20020370 **C** NHMUK 20020375, paratype1 **D** NHMUK 20020388 **E** NHMUK 20020374 **F** NHMUK 20020375, paratype2 **G** NHMUK 20020375, paratype3 **H** NHMUK 20020372 **I** NHMUK 20020381 **J** NHMUK 20020378 **K** NHMUK 20020382 (holotype) **L** NHMUK 20020380 **M** NHMUK 20020376.

**Figure 3.**
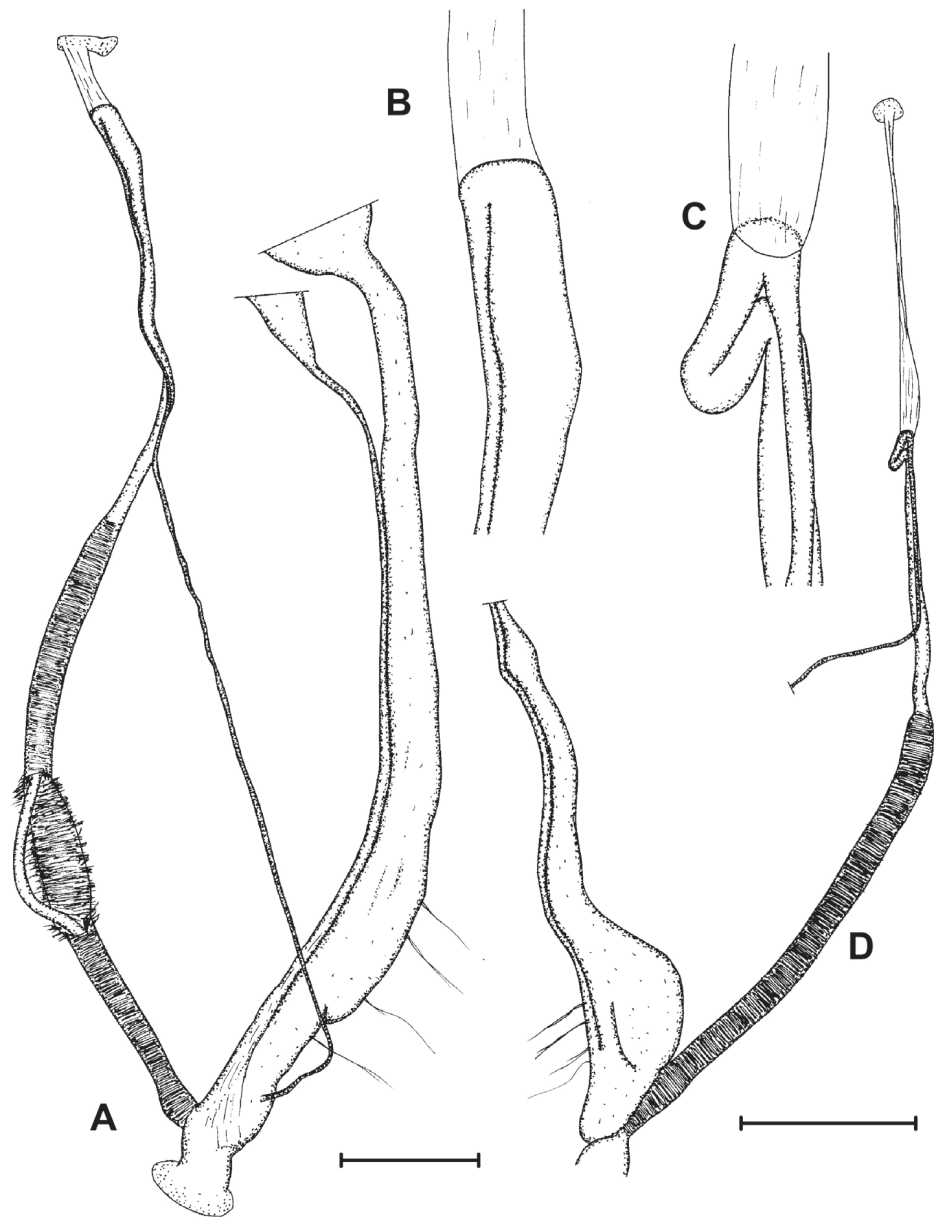
Genital anatomy of a paratype (NHMUK 20020422) of *Zilchistrophia hilaryae* sp. n. (**A–B**) (penis partly removed from its tunica), and a paratype (NHMUK 20020421) of *Zilchistrophia shiwiarorum* sp. n. (**C–D**). **B** and **C** show the epiphallus-vas deferens transition enlarged. Scales represent 1 mm, and refer to **A** and **D**.

**Figure 4.**
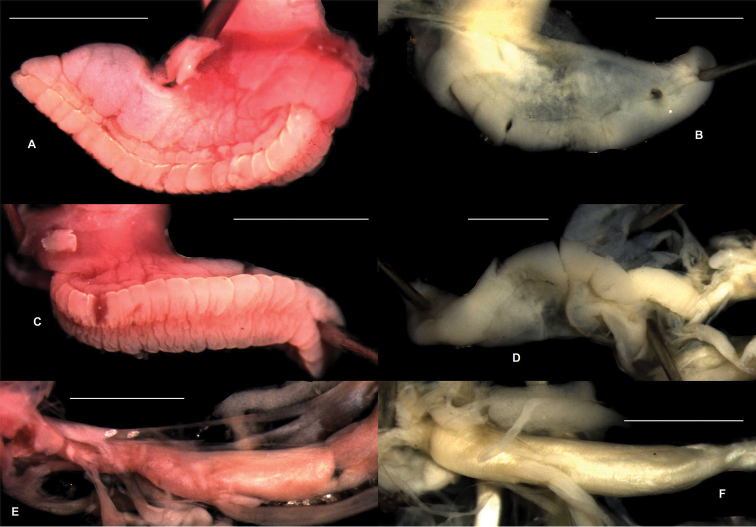
Lateral (**A–B**) and basal (**C–D**) side of the foot and buccal mass (**E–F**) of *Zilchistrophia shiwiarorum* sp. n. **A, C, E** (paratype, NHMUK 20020421) and *Zilchistrophia hilaryae* sp. n. **B, D, F** (paratype, NHMUK 20020422). Scales represent 1 mm.

**Figure 5.**
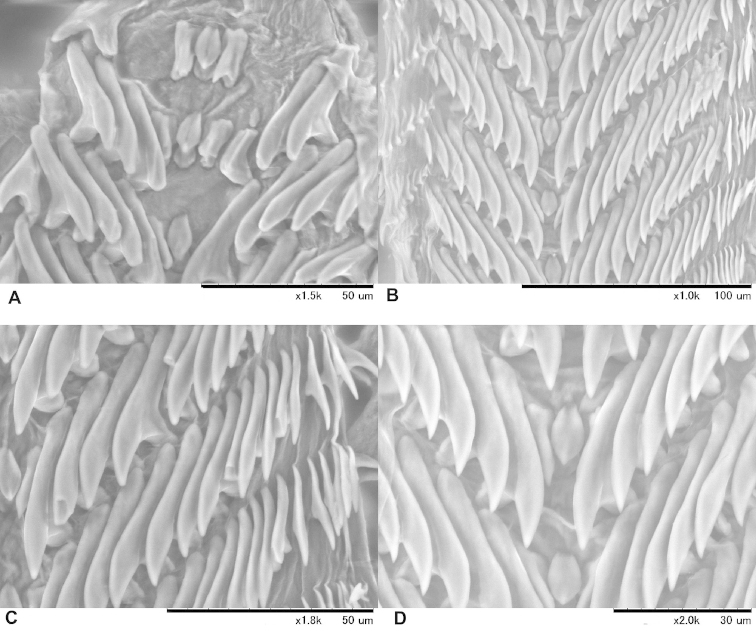
Radula of *Zilchistrophia hilaryae* sp. n. (NHMUK 20020422) **A** Central region at the terminal portion of the radula, **B** and **D** central region **C** lateral teeth.

Figs 1B–C, E , 2A–H , 3A–B , 4B, D, F , 5A–D

#### Type material.

Ecuador, Pastaza Province, Chuintsa, transect 7, (samples 360–369), leg. Hilary Kingston, 17.09.2000., NHMUK 20020375.1 (holotype), NHMUK 20020375.2–10 (9 paratypes); Ecuador, Pastaza Province, Nuevo Corrientes, transect 6, 02°00.224'S, 076°45.712'W (sample 175), leg. Hilary Kingston, 11.09.2000., NHMUK 20020384/1 paratype; Ecuador, Pastaza Province, Nuevo Corrientes, transect 6, 02°00.224'S, 076°45.712'W (sample 163), leg. Hilary Kingston, 11.09.2000., NHMUK 20020385/1 paratype; Ecuador, Pastaza Province, Chuintsa, transect 7, (sample 426b), leg. Hilary Kingston, 17.09.2000., NHMUK 20020372/1 paratype; Ecuador, Pastaza Province, Chuintsa, transect 7 (samples 419–425), leg. Hilary Kingston, 17.09.2000., NHMUK 20020374/7 paratypes; Ecuador, Pastaza Province, Chuintsa, transect 7, (samples 461–466), leg. Hilary Kingston, 17.09.2000., NHMUK 20020370/6 paratypes; Ecuador, Pastaza Province, Chuintsa, transect 7, (sample 305), leg. Hilary Kingston, 17.09.2000., NHMUK 20020387/1 paratype; Ecuador, Pastaza Province, Nuevo Corrientes, (sample 200), leg. Hilary Kingston, 13.09.2000., NHMUK 20020388/1 paratype; Chuintza, Pastaza, Ecuador, sample 308, Tissue sample J5, leg. Hilary Kingston, 17.09.2000., NHMUK 20020422 (dissected, ethanol-preserved animal).

#### Diagnosis.

A small *Zilchistrophia* species with regularly growing whorls, rounded body whorl, relatively wide umbilicus and three palatal plicae approximately a half whorl behind the aperture. The uppermost two plicae are situated very close to each other, forming a single-looking plica.

#### Description of the shell

(Figs 1–2): Shell dextral, yellowish, glossy and translucent, smooth, only irregular, very fine growth lines can be seen; shell shape discoid, with slightly domed apical surface; whorls 6 (n=4), regularly growing, the last whorl and especially the apertural part is conspicuously wider than the penultimate whorl; body whorl rounded; whorls are separated by relatively deep suture; umbilicus relatively wide, funnel-shaped; aperture crescent-shaped, with slightly thickened peristome; parietal callus not conspicuous, present as slight, blunt thickening, its sculpture is extremely finely granulated, rather matt; in corroded shells the callus is whitish, whereas the penultimate whorl can remain translucent (in those shells the callus is better visible).

One, two or three sets of plicae are situated in various positions behind the aperture (see Fig. 2 and remarks). One set consists of three horizontal, short palatal plicae. The first two plicae are very close to each other, forming a single-looking plica.

**Measurements (in mm).** D: 4.1–5.0, H: 2.0–2.4 (n=3).

#### Description of the anatomy.

One specimen was anatomically examined (Chuintza, Pastaza, Ecuador, sample 308, Tissue sample J5, leg. Hilary Kingston, 17.09.2000., NHMUK 20020422).

**Body.** Foot seemingly holopod, but it was laterally very much depressed (probably also decayed internally), therefore the real morphology could not be clearly examined (Fig. 4B, 4D); caudal horn absent, jaw absent, buccal mass conspicuously elongated (Fig. 4F); the pallial complex could not be examined due to the decay of the body.

**Radula** (Fig. 5). Long and narrow; central tooth small, pointed oval; the central and first lateral teeth are clearly separated; lateral teeth dagger-like, 19 in number on each side in each V-shaped row; the curved cusps of the lateral teeth point toward the centre and are connected by an extension to the basal figs that point away from the centre; first lateral tooth similar in shape to the other laterals, and it is conspicuously smaller than the second lateral tooth.

**Genitalia** (Fig. 3A–B). The right ommatophoral retractor runs between penis and vagina; penis long, slender, simple thin-walled tube, without any notable inner structure; penis surrounded by a thick, fibrous tunica; the end point of the penis is considered where the tunica narrows; epiphallus slightly shorter and slimmer than the penis (including the tunica), although tapers until proximal end; the short retractor muscle inserts on the epiphallus-vas deferens transition; vas deferens enters epiphallus subapically, slender, it is attached to the epiphallus almost along the complete length of the epiphallus; atrium relatively long, internally with fine longitudinal sculpture; vagina very short, it is attached to the body wall with a few fibres; inner wall of vagina finely reticulated; spermoviductus with swollen distal part with folded/reticulated inner surface; no embryos were found within the uterus; the distal end of the stalk of the bursa copulatrix forms a sheath which partly covers the vagina; the bursa copulatrix and the posterior part of the spermoviductus could not be investigated because the decayed condition of the examined specimen.

#### Differential diagnosis.

*Zilchistrophia hilaryae* sp. n. differs from *Zilchistrophia shiwiarorum* sp. n. by the larger size, weaker peristome, wider umbilicus, the rounded body whorl and the upper plica which consists of two joint plicae. Moreover, *Zilchistrophia shiwiarorum* sp. n. has more regularly growing whorls, (the apertural part is wider in *Zilchistrophia hilaryae* sp. n. from dorsal view). There are some differences in the anatomy between the two Ecuadorian species, such as the length of the retractor muscle and the presence or absence of the hook of the proximal epiphallus, although more material is needed to see if these represent reciprocally stable characters.

All three Peruvian *Zilchistrophia* species have more whorls than the Ecuadorian ones, and the area just behind the peristome margin conspicuously inflated, whereas this part is not inflated in the two new Ecuadorian species. The umbilicus of all three Peruvian species is regularly funnel-shaped with the last quarter of whorl being more far from the preceding whorl from ventral view.

Addition to this difference, The Peruvian species are larger than *Zilchistrophia hilaryae* sp. n. and have narrower umbilicus. Moreover, *Zilchistrophia tridentata* has three short palatal plicae in equal distance between each other. See also remarks.

#### Etymology.

*Zilchistrophia hilaryae* sp. n. is dedicated to Mrs. Hilary May (maiden name: Kingston), who collected the snails during the expedition to Ecuador.

#### Type locality.

Ecuador, Pastaza Province, Chuintsa.

#### Distribution

**Figure 6.**
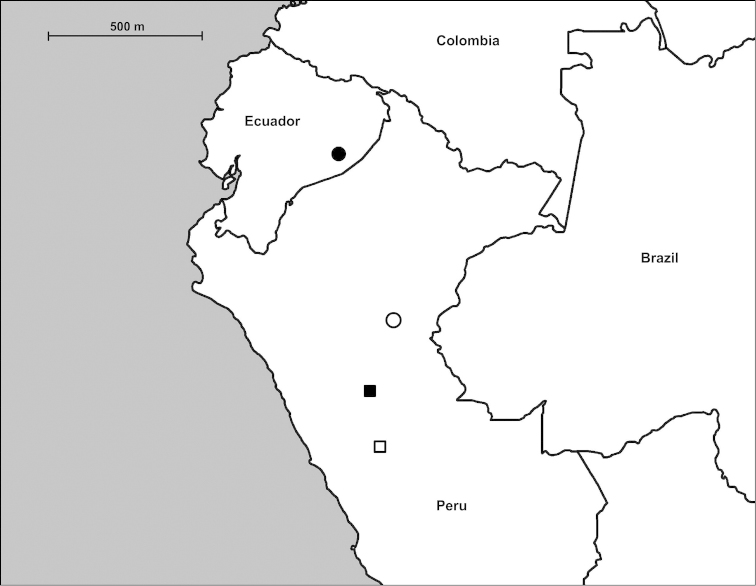
Distribution of *Zilchistrophia* Weyrauch, 1960 species. Filled circle: *Zilchistrophia hilaryae* sp. n. and *Zilchistrophia shiwiarorum* sp. n.; empty circle: *Zilchistrophia obvoluta* (Haas, 1949); filled square: *Zilchistrophia angigyra* (Haas, 1949); empty square: *Zilchistrophia tridentata* Weyrauch, 1960.

(Fig. 6). *Zilchistrophia hilaryae* sp. n. is known only from the vicinity of Chuintsa and Nuevo Corrientes, Pastaza Province, Ecuador.

#### Ecology.

The snails were collected among the leaf litter in open areas on the floor of the rain forest.

#### Conservation status.

Like in case of all other species inhabiting rain forest ecosystems, the main threats are deforestation and disturbance of the natural forests. The Shiwiar tribe has already called international attention, volunteering programs are focusing on them. We might assume that this would be positive in the conservation of the rain forest inhabited by the Shiwiar tribe.

#### Remarks.

The plicae can be observed in case of fresh, translucent shells only. Although in some corroded shells a single set of plicae is visible behind the aperture, we have not depicted them on Fig. 2, because additional sets of plicae may be present deeper in the shell, which are invisible due to the non-transparent shell wall. Even in crystal clear shells there might be plicae other than in the last whorls, but these cannot be observed without breaking the shell. Regardless of the difficulties in observing the inner plicae, it seems that juvenile shells have three sets of plicae, approximately a third whorl distance between each other. We assume that the previous sets of plicae (or some of them) are dissolved during growth.

We examined the inner morphology of the plicae in a subadult shell by breaking a part of less than a quarter of whorl off (Fig. 1C). The upper plica (=plica closer to the upper suture) was “double”, by having the upper and lower edges more elevated than its middle portion, and the lower plica (=plica closer to the lower suture) was “simple”. The shell of the anatomically examined specimen had to be broken, which offered the possibility to examine the inner side of the palatal wall. The upper plica in fact consists of two plicae which are situated close to each other and are in contact (Fig. 1E). This results the strange concave shape of the upper plica from apertural view.

A part of the shell of the paratype of *Zilchistrophia tridentata* bearing two of the palatal plicae is deposited in the Senckenberg Museum (SMF 162900). On that shell fragment both of the plicae were simple.

### Zilchistrophia shiwiarorum

Taxon classificationAnimaliaStylommatophoraScolodontidae

Páll-Gergely sp. n.

http://zoobank.org/7B0E714B-82E9-4DAC-803C-D9F1E02E1FD4

Figs 1D, F , 2I–M , 3C–D , 4A, C, E

#### Type material.

Ecuador, Pastaza Province, near Nuevo Corrientes, transect 5 (sample 141), leg. Hilary Kingston, 08.09.2000., NHMUK 20020382/1 holotype; Ecuador, Pastaza Province, near Nuevo Corrientes, transect 5 (sample 137), leg. Hilary Kingston, 08.09.2000., NHMUK 20020381/1 paratype; Ecuador, Pastaza Province, near Nuevo Corrientes, transect 5 (sample 101), leg. Hilary Kingston, 08.09.2000., NHMUK 20020378/1 paratype; Ecuador, Pastaza Province, near Nuevo Corrientes, transect 5 (sample 128), leg. Hilary Kingston, 08.09.2000., NHMUK 20020380/1 paratype; Ecuador, Pastaza Province, near Nuevo Corrientes, transect 5 (sample 125), leg. Hilary Kingston, 08.09.2000., NHMUK 20020379/1 paratype; Ecuador, Pastaza Province, near Nuevo Corrientes, transect 5 (sample 143), leg. Hilary Kingston, 08.09.2000., NHMUK 20020383/1 paratype; Ecuador, Pastaza Province, valley south of Nuevo Corrientes, transect 3, 02°00.365'S, 076°45.933'W, 321 m asl. (sample 37), leg. Hilary Kingston, 01.09.2000., NHMUK 20020368/1 paratype; Ecuador, Pastaza Province, valley south of Nuevo Corrientes, transect 3, 02°00.365'S, 076°45.933'W, 321 m asl. (sample 39), leg. Hilary Kingston, 01.09.2000., NHMUK 20020377/1 paratype; Ecuador, Pastaza Province, Nuevo Corrientes (sample 90), leg. Hilary Kingston, 05.09.2000., NHMUK 20020376/1 paratype; Transect 3, forest floor near Nuevo Corrientes, Pastaza, Ecuador, sample 33, leg. Hilary Kingston, 01.09.2000., NHMUK 20020421 (dissected, ethanol-preserved animal).

#### Diagnosis.

A small *Zilchistrophia* species with regularly growing whorls, angled body whorl, narrow umbilicus and two palatal plicae approximately half a whorl behind the aperture.

#### Description of the shell.

Shell dextral, whitish, glossy and translucent, smooth, only irregular, very fine growth lines can be seen; shell shape discoid, with domed apical surface; whorls 6.5 (n=2), regularly growing, only the apertural part is slightly wider than the penultimate whorl; body whorl with blunt but conspicuous upper keel; whorls are separated by relatively deep suture; umbilicus narrow, regular funnel-shaped; aperture deformed crescent-shaped (because of the upper keel), with thickened peristome; parietal callus not conspicuous, present as slight, blunt thickening, its sculpture is extremely finely granulated, rather matt.

One, two or three sets of horizontal, short plicae are situated behind the aperture (Fig. 2I–M). Both plicae are simple, horizontal thickenings on the parietal wall (Fig. 1F). These plicae can be observed in the case of fresh, translucent shells only. A juvenile shell had three sets of plicae, whereas all adult shells had one or two sets of plicae only. In this species as well, we assume that the previous sets of plica are in most cases dissolved during growth. See also remarks under *Zilchistrophia hilaryae* sp. n.

**Measurements (in mm).** D: 3.5–3.9, H: 2.2–2.5 (n=4).

#### Description of the anatomy.

One specimen was anatomically examined (Transect 3, forest floor near Nuevo Corrientes, Pastaza, Ecuador, sample 33, leg. Hilary Kingston, 01.09.2000., NHMUK 20020421).

**Body.** The part of the body which filled the last whorl of the ethanol-preserved specimen had an intensive pink/orange colour, whereas the ethanol was slightly pinkish. The remaining parts of the animal were brown. It is unknown whether this was the original colour of the living specimen, or it is the result of a secondary chemical reaction. Foot clearly aulacopod (Fig. 4C); caudal horn absent, but there is a seemingly inflated thickening above the tail in the ethanol-preserved animal (probably glandula, see Fig. 4A); jaw absent; buccal mass conspicuously long (Fig. 4E); the pallial complex could not be examined due to the decay of the body.

**Radula.** Indistinguishable from that of *Zilchistrophia hilaryae* sp. n.

**Genitalia** (Fig. 3C–D). The right ommatophoral retractor runs between penis and vagina; penis long, slender, simple thin-walled tube, without any notable inner structure; penis surrounded by a thick, fibrous tunica; the end point of the penis is considered where the tunica narrows; epiphallus is approximately half of the size of the penis, and it is more slender than the penis including the tunica; the proximal end of the epiphallus forms a loop before the insertion of the retractor muscle; the long retractor muscle is thickened distally, and inserts on the epiphallus-vas deferens transition; vas deferens slender, enters epiphallus subapically, it is attached to the epiphallus almost along the complete length of the epiphallus; vagina very short, it is attached to the body wall with a few fibres; the thickened part of the spermoviductus is in fact a cavity which joins the rest of the inner space through a narrowing; no embryos were found within the uterus; the bursa copulatrix and the posterior part of the spermoviductus could not be investigated because the decayed condition of the body.

#### Differential diagnosis.

*Zilchistrophia shiwiarorum* sp. n. can be distinguished from the other four species by the small shell size and the angled body whorl. See also the differential diagnosis under *Zilchistrophia hilaryae* sp. n.

#### Etymology.

*Zilchistrophia shiwiarorum* sp. n. is named after the Shiwiar tribe, which inhabits the area where both new species live.

#### Type locality.

Ecuador, Pastaza Province, near Nuevo Corrientes.

#### Distribution

(Fig. 6). *Zilchistrophia shiwiarorum* sp. n. is known only from the vicinity of Nuevo Corrientes, Pastaza Province.

#### Ecology.

Same as in *Zilchistrophia hilaryae* sp. n.

#### Conservation status.

See under *Zilchistrophia hilaryae* sp. n.

## Discussion

### Systematic position of *Zilchistrophia*

*Zilchistrophia* is the member of the Scolodontidae based on the reduced jaw, the aulacopod foot, and the dagger-like lateral teeth with basal figs, which point away from the centre. The small central tooth, the first lateral tooth which is considerably smaller than the second, the lack of a caudal horn, and the position of the right ommatophoral retractor, which passes between penis and vagina, indicate that *Zilchistrophia* belongs to the subfamily Scolodontinae (=Systrophiini Thiele, 1920, see Tillier 1980 and Schileyko 2000). Other genera of Scolodontinae are the following: *Entodina* Ancey, 1887, *Drepanostomella* Bourguignat, 1889, *Guesteria* Crosse, 1872, *Happia* Bourguignat, 1889, *Hirtudiscus* Hylton Scott, 1973, *Systrophia* L. Pfeiffer, 1855, and *Systrophiella* Baker, 1925. In contrast, the other subfamily of Scolodontidae, Tamayoinae Tillier, 1980 (including the genera, *Happiella* Baker, 1925, *Prohappia* Thiele, 1927, *Tamayoa* Baker, 1925 and *Tamayops* Baker, 1928) is characterized by the presence of a caudal horn, their ommatophoral retractor passes outside the peni-oviducal angle, have a more developed central tooth, and the first lateral tooth is not larger than the second one. *Drepanostomella* and *Hirtudiscus* were placed in the Tamayoini and the Endodontidae Pilsbry, 1895 (Punctoidea), respectively (Tillier 1980, Hylton Scott 1973), but both were transferred to the Scolodontinae by Hausdorf (2003). *Guesteria* was moved to the Scolodontinae by the unpublished work of Ramírez (1993) (see also Cuezzo and Miranda 2009).

The genera *Hirtudiscus* Hylton Scott, 1973 and *Drepanostomella* probably form a distinct subgroup within Scolodontinae by their peculiar suture, an incision at the parietal angle of the aperture, the general shell shape, the morphology of the inner structure of the penis (papillae with corneous hooks) and the presence of hairs and other periostracal structures on the shell (Hausdorf 2003, Cuezzo and Miranda 2009). *Guesteria* Crosse, 1872 is probably also a relative of *Hirtudiscus* and *Drepanostomella*, although its anatomy is insufficiently known. These three genera are probably only distantly related to *Zilchistrophia*. Interestingly, the number of teeth (19) is the same in *Hirtudiscus* and *Drepanostomella* and *Zilchistrophia*, but the above mentioned anatomical differences suggest that this trait is probably a coincident.

The genital anatomy of *Zilchistrophia* is also similar to those of most of the genera of Scolodontinae sensu Tillier (1980) (with the exception of *Hirtudiscus*, *Drepanostomella* and *Guesteria*) by the simple penis with no notable inner structure and a thick outer tunica, the vas deferens which enters the epiphallus subterminally, the retractor muscle which attaches on the proximal end of the epiphallus and the short vagina.

The genital anatomy of *Happia* is not known, but Schileyko (2000) assumed close relationship between *Happia* and *Systrophiella* by classifying the latter as the subgenus of the former. *Zilchistrophia* differs from *Happia* sensu Schileyko (2000) by the absence of circular fascia at the proximal end of the penis, the presence of a penial tunica, the lack of a globular penial caecum near the distal end of the penis, the fewer lateral teeth and the relatively larger central tooth. The vas deferens of *Scolodonta* (see Hausdorf 2006) is not attached to the epiphallus (attached in *Zilchistrophia*), but is connected to the proximal end of the penial tunica (similarly to the circular fascia in *Systrophiella*). Moreover, *Scolodonta* has shorter and more muscle fibres attaching the vagina to the body wall, it has relatively shorter epiphallus, longer vagina and fewer teeth in the radula. The seemingly closest relative of *Zilchistrophia* in terms of genitalia is *Wayampia* Tillier, 1980 (originally described as the subgenus of *Systrophia*), which also lacks the penial caecum, and its vas deferens is attached to the epiphallus. On the other hand, *Wayampia* has more rows of teeth and possess a thin jaw, which was not found in the two *Zilchistrophia* species. The anatomy of *Entodina* is unknown, but it is similar to *Zilchistrophia* in possessing a shell with slightly thickened peristome; a trait which is possibly a synapomorphy of these two genera. *Entodina* differs from *Zilchistrophia* by the smaller number of lateral teeth, the flatter shell, the absence of the palatal denticles behind the aperture, and by the presence of palatal tubercle on the peristome.

Conchologically *Zilchistrophia* differs from all other members of Scolodontinae by the presence of two or three palatal plicae. The taxonomic relationship between the Peruvian and Ecuadorian *Zilchistrophia* is questionable without knowing the anatomy of Peruvian species. Peruvian species have an inflated last quarter of whorl, whereas this part is not conspicuously widened in Ecuadorian species.

### Relationship with Plectopyloidea

*Zilchistrophia* species have a typical systrophiid appearance (translucent, glossy, sculptureless, flat shells with several slowly growing whorls and crescent-shaped, toothless aperture. The two or three short, horizontal plicae, which are situated approximately a third to half whorl behind the aperture is unusual in the family. *Zilchistrophia* was provisionally classified within the Corillidae because of the “striking” similarity between its palatal plicae with those of the genus *Plectopylis* (see Weyrauch 1960). Weyrauch probably referred to Corillidae in the understanding of Zilch (1959–1960), according to which, four genera belong to the Corillidae: the Chinese *Amphicoelina* Haas, 1933, the Sri Lankan and South Indian *Corilla* H. & A. Adams, 1885, the East Asian *Plectopylis* Benson, 1860 and the African *Sculptaria* L. Pfeiffer, 1855. In the classification of Bouchet et al. (2005), Corillidae, Plectopylidae and Sculptariidae form the superfamily Plectopyloidea, whereas in Schileyko’s (2000) classification, which is followed here, Sculptariidae is placed in the superfamily Acavoidea Pilsbry, 1894, and Corillidae and Plectopylidae form the superfamily Plectopyloidea. Although the anatomy of *Amphicoelina* is unknown, it rather belongs to Camaenidae, not to Plectopyloidea (see Páll-Gergely and Asami 2014). Corillidae is a monotypic family, whereas Plectopylidae includes seven genera, such as *Chersaecia* Gude, 1899, *Endoplon* Gude, 1899, *Endothyrella* Gude, 1899, *Gudeodiscus* Páll-Gergely, 2013, *Sicradiscus* Páll-Gergely, 2013, *Sinicola* Gude, 1899 and *Plectopylis* Benson, 1860 (Schileyko 1999, Páll-Gergely and Hunyadi 2013). Both Corillidae and Plectopylidae possess palatal plicae which are situated at most a half whorl behind the peristome. These palatal plicae rarely visible from the aperture, but they never reach the peristome. *Corilla* primarily have four or five long, horizontal or oblique palatal plicae, which are reduced to one or zero in two species. Plectopylidae primarily possess six short horizontal palatal plicae (5 or 7 in few species), which are modified in many species (united to each other or divided in the middle, the middle plicae are often oblique, see Gude 1914 and Páll-Gergely and Hunyadi 2013, and references therein). Some *Sicradiscus*, *Sinicola* and *Gudeodiscus* species (e.g. *Gudeodiscus multispira* [Möllendorff, 1883]) certainly show strong resemblance to *Zilchistrophia* species in terms of the shell size, glossy surface and toothless aperture. The main conchological difference between *Zilchistrophia* and those Asian families is that in the Corillidae there are horizontal plicae, and in the Plectopylidae there are horizontal plicae as well as vertical lamellae on the parietal side. Parietal plicae or lamellae are entirely missing in *Zilchistrophia*. Moreover, most plectopylid genera have finely ribbed embryonic whorls, which are sculptureless in *Zilchistrophia*.

The overall genital structure of *Zilchistrophia* and Plectopylidae may look similar because both groups have “simple” reproductive organs lacking dart sacs, glandulae, etc. The main differences are the following: Plectopylidae lack the penial tunica which is well-developed in *Zilchistrophia*; the inner wall of the penis of Plectopylidae is complicated, usually with longitudinal or reticulated, often with calcareous granules, whereas in *Zilchistrophia* there is no penial sculpture visible; in *Zilchistrophia* the retractor muscle inserts on the epiphallus-vas deferens transition, but in Plectopylidae it inserts on the penial caecum, or if the caecum is missing, than on the penis-epiphallus transition; the vagina of *Zilchistrophia* is very short, but relatively long in Plectopylidae, usually with a “vaginal bulb” in the middle; most Plectopylidae have a diverticulum which originates from the wall of the pedunculus, but *Zilchistrophia* probably lacks a diverticulum, or at least it does not originate from the wall of the pedunculus (see Stoliczka 1871, Schileyko 1999, Páll-Gergely and Hunyadi 2013 and references therein).

The genitalia of Corillidae mainly differs from that of *Zilchistrophia* in the following: penial tunica missing (well-developed in *Zilchistrophia*); retractor muscle inserts on the middle of the epiphallus (on the epiphallus-vas deferens transition in *Zilchistrophia*), and penial papilla well-developed (not found in *Zilchistrophia*).

In the molecular phylogeny published by Ramírez et al. (2012) Systrophiidae represent the “third stylommatophoran clade” next to the “achatinoid” and “non-achatinoid” clades defined by Wade et al. (2006). Their results also confirm that the morphological similarities between *Zilchistrophia* and *Corilla* (member of the “non-achatinoid-clade”), especially the presence of palatal plicae can be explained by parallel evolution. Plectopylidae were not used in the molecular phylogeny of these studies, but its position is expected to be similar to that of the genus *Corilla*.

## Supplementary Material

XML Treatment for Scolodontidae

XML Treatment for Zilchistrophia

XML Treatment for Zilchistrophia hilaryae

XML Treatment for Zilchistrophia shiwiarorum
